# A Comparative Study Between Copy Number Alterations and PRAME Immunohistochemical Pilot Study in Challenging Melanocytic Lesions

**DOI:** 10.3390/cancers17071218

**Published:** 2025-04-04

**Authors:** Jeana Chun, Ashley R. Scholl, Jennifer Crimmins, Michelle M. Schneider, M. Angelica Selim, Rami N. Al-Rohil

**Affiliations:** 1 School of Medicine, Duke University, Durham, NC 27710, USA; 2 Department of Pathology, Duke University Medical Center, Durham, NC 27710, USA; ashley.scholl@duke.edu (A.R.S.); jennifer.crimmins@duke.edu (J.C.); michelle.schneider@duke.edu (M.M.S.); angelica.selim@duke.edu (M.A.S.); 3 Department of Dermatology, The University of North Carolina Chapel Hill, Chapel Hill, NC 27599, USA

**Keywords:** melanoma, nevus, PRAME, copy number alteration, diagnostically challenging

## Abstract

Introduction: Diagnostic uncertainty for ambiguous lesions that fall on the spectrum between nevi and melanoma remains a significant challenge and can have consequences for patient management. Methods: This study aimed to compare the diagnostic utility of preferentially expressed antigen in melanoma (PRAME) immunohistochemistry to molecular testing (FISH and SNP array) in 34 diagnostically challenging melanocytic lesions and 9 non-diagnostically challenging melanomas. Results: We conclude that while PRAME immunohistochemistry demonstrates high specificity (96.2%) in diagnostically challenging melanocytic lesions, its low sensitivity (12.5%) suggests that it should not replace histopathological evaluation in rendering the final diagnosis. Conclusions: These findings suggest that PRAME may serve as a useful adjunct in the diagnostic workup, particularly due to its high negative predictive value, but should be used in conjunction with other established diagnostic modalities.

## Simple Summary

Melanoma is a significant public health concern due to its rising incidence and mortality. While most melanocytic lesions can be readily classified as benign nevi or malignant melanoma, diagnostically challenging lesions remain difficult to categorize due to their overlapping clinical and histological features. Ancillary tools such as immunohistochemistry, FISH, and SNP array have been developed to enhance diagnostic accuracy. This study evaluates the diagnostic performance of PRAME, a tumor-associated antigen, to molecular testing in 34 diagnostically challenging and 9 non-diagnostically challenging melanomas. In diagnostically challenging cases, PRAME showed high specificity (96.2%) and negative predictive value (78.1%) but low sensitivity (12.5%), whereas molecular testing demonstrated higher sensitivity (75%) and specificity (100%). These findings suggest that while the utility of PRAME is limited as a standalone test, it does serve as a useful confirmatory adjunct, especially when a benign diagnosis is favored.

## 1. Introduction

Melanoma represents a significant public health concern due to its increasing incidence and associated mortality rates. According to the American Cancer Society, there will be 100,640 new cases of melanoma and 8290 deaths due to melanoma in 2024 [1]. Risk factors for melanoma include excessive exposure to ultraviolet radiation, personal or family history of melanoma, the presence of multiple nevi or atypical nevi, fair skin complexion, and weakened immune system [2].

When a pigmented lesion is encountered with clinically atypical or concerning features, histopathologic evaluation remains the gold standard in determining its biologic potential. Accurate diagnosis represents a critical fork in clinical management. Atypical nevi can be monitored or conservatively excised; however, melanomas are excised with larger margins and possibly undergo sentinel lymph node sampling to control the disease, stratify the risk of the disease, and determine the need for additional medical intervention [3].

Most melanocytic lesions are readily classified as melanoma or nevi (including atypical nevi) on histopathologic evaluation. However, the characteristics distinguishing melanomas from severely atypical nevi are not binary but exist on a spectrum. Because of this, melanocytic lesions that demonstrate ambiguous histological and clinical findings pose a diagnostic challenge. Among these diagnostically challenging melanocytic lesions, there is a lack of standardization and accuracy in classification [4].

This ambiguity highlights the need for reliable diagnostic biomarkers to aid in the detection of diagnostically challenging melanoma. Techniques such as immunohistochemistry (IHC), fluorescence in situ hybridization (FISH), genome-wide array comparative genomic hybridization (CGH), and single nucleotide polymorphism (SNP) through the years have emerged as useful tools to help predict the biological potential in diagnostically challenging melanocytic lesions [5,6,7,8].

One tumor-associated immunohistochemical antigen that has garnered recent interest as a potential diagnostic and prognostic marker for melanoma is preferentially expressed antigen in melanoma (PRAME) [9,10]. This antigen represses retinoic acid receptor signaling, which typically protects against tumorigenesis by increasing the expression of genes for growth arrest, cell death, and differentiation [11]. PRAME is differentially methylated in malignant tissue compared to normal tissue [12]. It is highly expressed via epigenetic hypermethylation in various cancers, including melanoma, but its expression in normal tissue is limited via hypomethylation to the testes, ovaries, placenta, and adrenal glands [13]. This selective expression in cancer tissue makes PRAME a promising target for cancer-specific diagnostics and therapeutics.

The utility of PRAME and its concordance with ancillary molecular testing in diagnostically challenging lesions have not been widely investigated. This study aims to compare PRAME’s diagnostic utility to molecular testing (FISH and SNP array), particularly in diagnostically challenging melanocytic lesions.

## 2. Materials/Methods

### 2.1. Case Selection

After institutional review board approval (Pro00115496), 43 cases of melanocytic lesions from the institution archives were identified as melanocytic lesions that underwent PRAME and molecular analysis between November 2018 and July 2022 and were identified in the electronic health record system Epic. Nine cases were non-diagnostically challenging melanomas, and thirty-four cases were diagnostically challenging melanocytic lesions. The final diagnosis for each case was rendered by a board-certified dermatopathologist, encompassing clinical information, histopathologic and immunohistochemical features, and molecular profile.

### 2.2. Data Collection

Information collected included diagnosis, pathologic stage, Breslow thickness, PRAME result, molecular studies results, date of diagnosis, age at the time of diagnosis, gender, anatomical location, molecular results (FISH or SNP alterations), metastases, recurrence, lymph node sampling, and whether excision was performed. PRAME immunostaining utilized the PRAME Rb monoclonal antibody DAB and Red ready-to-use antibody by BioCare, catalog ALI 3252 G7. It was applied using bond immunohistochemical stainer on paraffin-embedded tissue cut at a 5 micron thickness. PRAME was considered positive when >75% of the lesion displayed nuclear staining. For copy number alterations, in cases tested by fluorescence in situ hybridization, at least 50 melanocytic nuclei were scored for copy number gains of 6p25 (RREB1)/centromere 6, 8q24 (MYC)/centromere 8, and 11q13 (CCND1)/centromere 11 or the homozygous deletion of 9p21 (CDKN2A)/centromere 9.

For each probe, a dual color interphase FISH assay was performed on paraffin-embedded sections of the tumor, and a positive result was defined as >29% of nuclei positive for any one of the above numerical abnormalities. SNP array testing on paraffin-embedded tissue in melanocytic tumors involves several key steps for DNA extraction after annotating the tumor area and macrodissecting the tumor cells from the unstained slides (at least 20% of the extracted area should contain tumor cells). Using a ThermoFisher Scientific microarray (Waltham, MA, USA), the extracted DNA is fragmented, labeled with fluorescent dyes, and hybridized to a microarray containing thousands of SNP probes that cover the entire genome. The microarray is scanned to detect fluorescent signals from the hybridized DNA. After these steps, the data are analyzed for fluorescence intensity to determine copy number alterations and the loss of heterozygosity across the genome. The copy number profile is examined for chromosomal gains, losses, and other alterations characteristic of melanoma. Typically, the presence of multiple copy number changes (>3) is considered indicative of malignancy. This technique enables the genome-wide detection of chromosomal abnormalities in melanocytic lesions, which can aid in distinguishing benign nevi from melanoma in diagnostically challenging cases. The ability to use FFPE tissue makes SNP array testing applicable to routine clinical specimens. For SNP array analysis, we used the criteria and methodology listed in Alomari et al., using a cutoff of >3 CNVs with some caveats, as the threshold for a positive result [14]. Follow-up information as of April 2024 was also collected. For patients alive as of April 2024, disease status was recorded. For patients who died before April 2024, the cause of death (melanoma or other) was recorded. Patient-identifying information was stored in the Protected Analytics Computing Environment (PACE).

### 2.3. Data Analysis

For data analysis, the diagnostically challenging melanocytic lesions were divided into those treated as melanoma and those treated as severely atypical nevi according to the final dermatopathologic diagnosis. The treatment for severely atypical nevi was excision with 3–5 mm margins. Cases diagnosed as melanoma were treated according to NCCN guidelines for melanoma management.

The concordance of PRAME results and the molecular results with the final diagnosis were calculated for the non-diagnostically challenging melanomas, all diagnostically challenging melanocytic lesions, and each subgroup of the diagnostically challenging melanocytic lesions (diagnostically challenging melanomas and severely atypical nevi).

Simple statistical analyses were then performed to calculate the sensitivity and positive predictive value of PRAME and molecular studies for the non-diagnostically challenging melanomas. Sensitivity, specificity, and positive and negative predictive values were calculated for the diagnostically challenging melanomas.

## 3. Results

Forty-three cases of melanocytic lesions were identified for data analysis (Table 1). Nine were classified as non-diagnostically challenging melanoma, while thirty-four were classified as diagnostically challenging melanocytic lesions. Eight of the thirty-four diagnostically challenging lesions were diagnosed as melanoma, while the other twenty-six were diagnosed as severely atypical nevi. Demographic details, location of lesions, staging and thickness of melanomas, as well as follow-up duration and information are presented in Table 2, Table 3 and Table 4. Within the category of diagnostically challenging cases that were diagnosed as severely atypical nevi included nineteen compound dysplastic nevi with severe atypia, three special site nevi with severe atypia, and four compound Spitz nevi with severe atypia. The cases that were diagnosed as melanoma included two nodular melanoma, two superficial spreading melanoma, two atypical spitz melanocytoma (which is worrisome for spitz melanoma), one acral lentiginous melanoma, and one melanoma with an associated intradermal nevus.

The copy number alterations and PRAME immunohistochemical results for all melanocytic lesions included in this study are summarized in Table 3. The copy number alterations were determined via either FISH testing or SNP array. Of the non-diagnostically challenging melanomas, 100% had copy number alterations and 77.8% were positive for PRAME. Of the diagnostically challenging melanocytic lesions, 17.6% had copy number alterations and 5.9% were positive for PRAME.

Table 4 presents the concordance values for PRAME and molecular studies for all melanocytic lesions. Copy number alteration concordance was 100% (100% sensitivity; 100% positive predictive value) for the non-diagnostically challenging melanomas. PRAME concordance for this category was 77.8% (77.8% sensitivity; 100% positive predictive value). For the diagnostically challenging melanocytic lesions, PRAME concordance was 76.5%, and molecular concordance was 94.1%. The data were further disaggregated to determine PRAME and molecular concordance for diagnostically challenging melanocytic lesions classified as severely atypical nevi vs. diagnostically challenging melanoma. The PRAME concordance and molecular concordance for the severely atypical nevi were 96.2% and 100%, respectively. The PRAME concordance and molecular concordance for diagnostically challenging melanoma were 12.5% and 75%, respectively.

Table 5 demonstrates the diagnostic accuracy measures of PRAME and molecular studies for diagnostically challenging lesions. The PRAME immunohistochemistry results for diagnostically challenging melanocytic lesions had a sensitivity of 12.5%, a specificity of 96.2%, a positive predictive value of 50%, and a negative predictive value of 78.1%. The molecular testing results for this category had a sensitivity of 75%, a specificity of 100%, a positive predictive value of 100%, and a negative predictive value of 92.9%.

**Figure 1:**
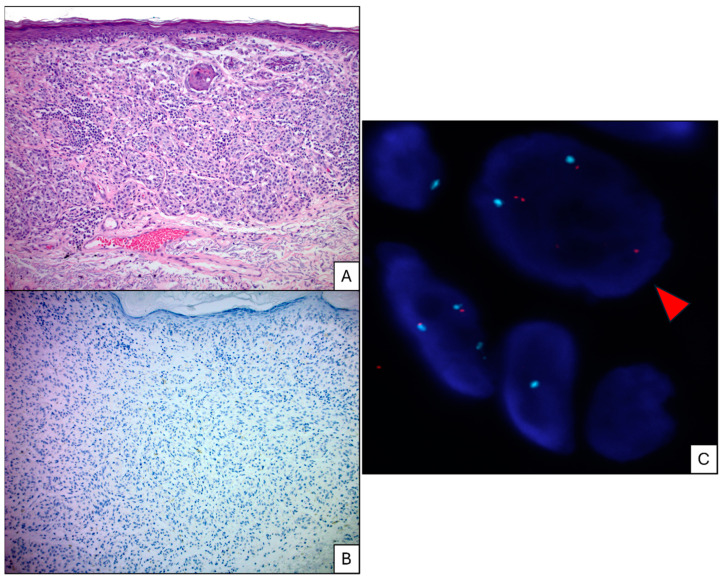
A clinically atypical, pigmented lesion from the left upper back of a 66-year-old female patient that shows significant architectural atypia. The junctional component shows confluence of single cells flattening of rete ridges and the dermal melanocytes grow in the form of expansile nests with associated non-brisk lymphocytic host response. The melanocytes show pleomorphism and no signs of maturation with descent ((**A**), H&E 100×). The melanocytes show no immunoreactivity for PRAME immunohistochemistry ((**B**), PRAME immunostain 100×). Fluorescence in situ hybridization showed significant gains in 6p25 (red dots) in comparison to centromere 6 (blue dots) and was seen in 32% of the examined melanocytes; the red arrows highlight two cells with gains ((**C**) fluorescence in situ hybridization 6p25 probe; 1000×).

Figure 1 demonstrates a case of diagnostically non-challenging melanomas that showed significant architectural and cytologic atypia with associated lymphocytic host response arising in sun-damaged skin; despite those atypical features, PRAME immunostaining was negative. Fluorescence in situ hybridization showed gains in 6p25 (*RREB1*).

**Figure 2:**
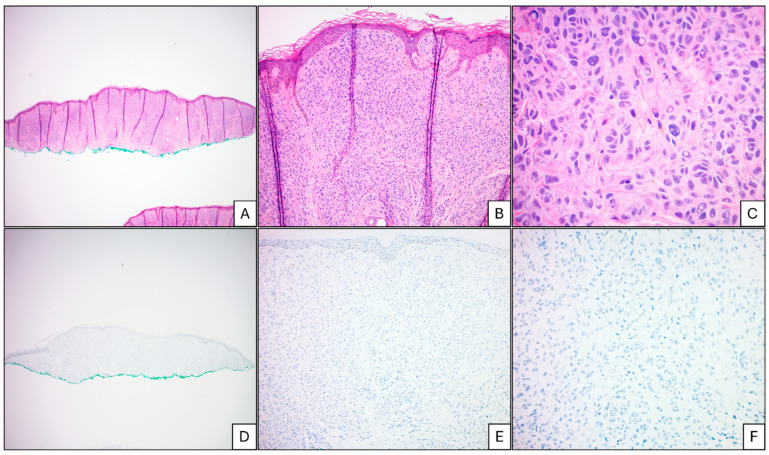
A non-pigmented melanocytic lesion on the forearm of a 47-year-old female patient with no previous history of melanoma. The melanocytic proliferation predominantly shows intradermal melanocytes ((**A**), H&E 40×). The nests grow in expansile nests with no signs of maturation ((**B**), H&E 100×). The melanocytes show marked pleomorphism with scattered large forms and nuclear inclusions; however, the background shows interspersed solar elastosis ((**C**), H&E 400×). PRAME immunostaining was negative throughout the lesion ((**D**), PRAME 40×; (**E**), PRAME 100×; (**F**) PRAME 400×). SNP array showed loss of one copy of entire chromosome 9 and X. Chromosome 1 q arm shows chromothripsis that consists of 22 copy number gains (3~4 copies) and 17 copy number losses (1 copy). The presence of chromothripsis is concerning for more aggressive biological behavior. The lesion was treated as a pT2a invasive melanoma and showed no residual disease and no evidence of metastatic melanoma in the sentinel lymph node. The patient is alive without disease at 61 months of follow-up.

Figure 2 demonstrates a diagnostically challenging melanocytic lesion that shows mostly dermal-based melanocytic proliferation growing in an expansile fashion with cytologic atypia and no signs of maturation. Dermal mitotic figures were not identified. There was no significant inflammatory host response. PRAME immunostaining was negative. SNP array demonstrated three copy number alterations along with chromothripsis, which was concerning regarding the biologic potential of this atypical melanocytic proliferation.

## 4. Discussion

Melanocytic lesions range from benign nevi to malignant melanomas, and within this spectrum are intermediate lesions that can pose significant diagnostic challenges. Determining the biological potential of diagnostically challenging lesions remains a pervasive problem in melanocytic pathology [15]. Multiple studies have documented the presence of discordance in the diagnoses of melanocytic lesions, even among experts in the field [16,17,18]. One such study presented expert pathologists with 30 unusual lymphoproliferative and melanocytic lesions. While the correct diagnosis was determined by 56% of the experts, the group came to a unanimous decision for only 7% of the lesions [18]. Furthermore, for 66% of the lesions, a final diagnosis required the analysis of histological, clinical, immunological, and molecular features. Taken together, the lack of concordance in the evaluation of diagnostically challenging melanocytic lesions among clinicians and the inability of a single diagnostic procedure to consistently determine a diagnosis highlight the need for integrating multiple diagnostic techniques and developing additional approaches.

PRAME immunohistochemistry is one such diagnostic technique that has emerged in recent years. In the seminal paper published in 2018, Lezcano et al. investigated PRAME expression in 400 melanocytic lesions classified as unambiguous nevi or melanoma [19]. They demonstrated that PRAME expression was positive in the majority of both primary and metastatic melanoma (83.2% and 87%, respectively) and negative in the majority of benign melanocytic nevi (86.4%) [19]. This differential expression of PRAME in melanoma has introduced PRAME immunohistochemistry as a promising ancillary diagnostic tool.

Other ancillary methods that supplement histopathological evaluation in distinguishing nevi from melanoma include molecular classification and copy number alteration. Molecular classification provides the genetic signature of melanocytic lesions via microarray gene expression profiling, and results have been shown to have high concordance rates with histopathology [20]. Furthermore, molecular studies have established that while nevi exhibit normal chromosomal complement, melanomas exhibit chromosomal losses, gains, and rearrangements [21]. This genetic instability in melanoma makes the evaluation of copy number alterations through fluorescent in situ hybridization (FISH) analysis a useful technique in enhancing the diagnostic accuracy in difficult melanocytic lesions. Previous studies have demonstrated this technique to have very high specificity and moderate-to-high sensitivity, supporting its value as an accompaniment to morphological assessment for challenging lesions [7,22].

The performance of PRAME immunohistochemistry in classifying diagnostically challenging tumors is a current focus of research. A recently published study reported that PRAME immunohistochemistry had comparable sensitivity to molecular classification via gene expression-profiling assay [23]. The agreement between PRAME and FISH and/or SNP has been less conclusive, with some studies reporting mild-to-moderate concordance and others reporting high concordance [10,24,25,26]. Another point of consideration when evaluating PRAME is the documented presence of diffuse positivity in spitzoid nevi, which are particularly problematic due to their widely varying and often ambiguous histopathological features [10,26]. This may undermine its usefulness as an adjunct diagnostic tool, although the current literature is mixed. Recently, Koh et al. showed that when staining intensity is taken into account in the interpretation of PRAME immunohistochemistry as positive or negative, PRAME may actually be a useful biomarker for distinguishing spitzoid melanomas from benign spitzoid neoplasms [27]. However, a subsequent study by Warbasse et al. comparing PRAME staining to FISH testing in 83 spitzoid melanocytic neoplasms found a low sensitivity of 29.6% [28]. Although the reported specificity was higher at 76.8%, the low sensitivity raises the risk of false negatives, which is concerning when considering PRAME as a potential screening test. Ultimately, this lack of consensus among studies demonstrates that more data are needed regarding the concordance of PRAME with established ancillary molecular testing in diagnostically challenging tumors.

Our study aimed to compare PRAME’s diagnostic utility to molecular testing (FISH and SNP array), particularly in ambiguous melanocytic lesions. The results showed that among diagnostically challenging melanocytic lesions, those classified as atypical nevi demonstrated a PRAME concordance and a molecular concordance of 96.2% and 100%, respectively. Lesions classified as diagnostically challenging melanoma showed a PRAME concordance and a molecular concordance of 12.5% and 75%, respectively.

For these diagnostically challenging lesions, PRAME immunohistochemistry results had a sensitivity of 12.5%, a specificity of 96.2%, a positive predictive value of 50%, and a negative predictive value of 78.1%. The molecular testing results for this category had a sensitivity of 75%, specificity of 100%, positive predictive value of 100%, and negative predictive value of 92.9%.

Regarding metastasis, one diagnostically challenging lesion from our study demonstrated sentinel lymph node metastasis but no further systemic metastasis in a 2-year-old. The tumor histologically showed atypical spitz cytomorphology with pleomorphism, atypia, and mitotic activity. PRAME immunostaining was negative. SNP array testing demonstrated intrachromosomal complexity (>5 distinct alternating copy number segments), resulting in multiple gains in different regions of 7q. The final diagnosis rendered was an atypical spitz melanocytoma with histologic and molecular alterations, which was worrisome for spitz melanoma. The patient had an isolated small-sized (<1 mm in greatest dimension) metastasis to one sentinel lymph node. However, the patient did not have any further systemic metastasis at the 3-month follow-up and was placed under observation. Future studies that assess the PRAME concordance in diagnostically challenging lesions with more unequivocal metastases will be interesting. In particular, further investigation into the utility of PRAME immunohistochemistry in cases with confirmed metastatic disease may help clarify its potential prognostic value. Understanding whether PRAME expression correlates with metastatic potential could offer insight into its role not only as a diagnostic tool but also as a marker of biological behavior.

Our results show that PRAME is as sensitive as molecular testing for classifying non-diagnostically challenging melanoma. However, it is significantly less sensitive than molecular testing for diagnostically challenging lesions. This low sensitivity of PRAME suggests that when conventional histopathological evaluation and established ancillary diagnostic tests indicate a diagnosis of melanoma for a given challenging lesion, the absence of PRAME expression alone should not be considered sufficient grounds to reclassify the lesion as benign. Conversely, the high specificity of PRAME suggests that when these established methods suggest a benign diagnosis, a negative PRAME result can be interpreted as a reassuring or confirmatory finding.

Furthermore, our findings add variance to recent studies investigating the agreement of PRAME immunohistochemistry with FISH and/or SNP and final diagnosis in diagnostically challenging melanocytic lesions. In an analysis of 110 challenging lesions, Lezcano et al. identified a 92.7% agreement between PRAME results and the final diagnostic interpretation [26]. Another analysis of 55 diagnostically challenging lesions reported PRAME sensitivity and specificity to be 91.7% and 93.5%, respectively [10]. Our comparatively low value of PRAME sensitivity (12.5%) is more in line with the aforementioned study by Warbasse et al. that reported a sensitivity of 29.6% [28]. However, our study adds the dimension of considering agreement with both FISH and SNP testing, as compared to only FISH testing, which was the case in the Warbasse study.

These results underscore the importance of integrating multiple diagnostic modalities rather than relying solely on a single biomarker. While PRAME immunohistochemistry serves as a useful adjunct in the diagnostic workup of melanocytic lesions, it should be used to complement, rather than override, the collective evidence from thorough histopathological examination and other well-established diagnostic techniques.

Limitations of this study include a small sample size of 43 melanocytic lesions, all taken from a single institution, as well as a short follow-up period. The limited sample size reflects the selective use of ancillary molecular testing such as FISH and SNP array in our dermatopathology practice. Melanocytic lesions at our institution are evaluated using histopathologic and immunohistochemical methods, and usually, a definitive diagnosis can be rendered without the need for further testing. Only a small subset of lesions—typically less than 1%—present significant diagnostic ambiguity warranting additional molecular analysis such as FISH or SNP array. PRAME immunohistochemistry is used more frequently than these molecular tests, but in this study, only cases that underwent both PRAME and molecular analysis were included. The nine non-ambiguous melanoma cases that underwent molecular testing in our sample size primarily did so for quality control purposes rather than for diagnostic purposes. The follow-up duration ranged between 21 and 65 months, with one of the cases within the diagnostically challenging category showing metastases. Additionally, the interpretation of PRAME staining percentage remains challenging as the 75% cut-off, regardless of the intensity of staining, may not always be reproducible; better standardization tools such as image analysis and artificial intelligence to make the staining interpretation standardized and unified may be beneficial.

In summary, our study demonstrates that PRAME is a useful tool that is particularly valuable for its high negative predictive value but should not replace the current gold standard of histopathological evaluation or the integration of results from multiple diagnostic modalities. Future research with a larger sample size and longer follow-up duration will help to more fully characterize the performance and utility of PRAME.

## Figures and Tables

**Table 1:** Demographics for all tested melanocytic lesions.

Demographics	
**Total Number of Cases**	43
**Age (years)**	
Mean	45.1
Range	2–92
**Gender**	
Male	18
Female	25
**Location**	
Trunk	23
Extremities	11
Head/Neck	9
**Non-Diagnostically Challenging Melanomas**	9
**Diagnostically Challenging Melanocytic Lesions**	34
Treated as Severely Atypical Nevi	26
Treated as Melanoma	8

**Table 2:** Clinical data and follow-up information for all tested melanocytic lesions.

	Non- Diagnostically Challenging Melanoma	Diagnostically Challenging Melanocytic Lesions Treated as Melanoma	Diagnostically Challenging Melanocytic Lesions Treated as Severely Atypical Nevus
**Total cases (** ***n*)**	9	8	26
**Age (years)**			
Range	66–92	2–69	15–61
Mean	77.89	38.1	36.7
Median	78	37.5	37
**Gender**			
Male	6	4	8
Female	3	4	18
**Location**			
Trunk	3	4	14
Extremities	4	4	6
Head/Neck	2	0	6
**Breslow** **thickness (mm)**			
Range	0.3–5.2	0.6–4.2	Na
Mean	1.63	1.74	Na
Median	0.95	1.49	Na
**Pathologic stage**			
**T**			
Range	1a–3b	1–4	Na
**N**			
Range	0–2c	0–1a	Na
**M**			
Range	0–1d	0	Na
**Follow-up duration (months)**			
Range	7–64	3–61	21–64
Mean	43.33	31.1	44.2
Median	50	28	51
**Follow-up information**			
Recurrent local disease	1	0	1
Metastatic melanoma	3	1	0
Alive with disease	3	8	26
Died of disease	2	0	0
Died of other causes	3	0	Na
Lost to follow up	0	0	0

**Table 3:** Copy number alterations and PRAME results for all tested melanocytic lesions.

Non-Challenging Melanomas (*n* = 9)	
**Copy Number Alterations (%)**	100%
Positive FISH (n)	9
Positive SNP array (n)	0
**PRAME Positivity (%)**	77.8%
Positive PRAME (n)	9
**Diagnostically Challenging Melanocytic Lesions (*n* = 34)**	
**Copy Number Alterations (%)**	17.6%
Positive FISH (n)	1
Positive SNP array (n)	5
**PRAME Positivity (%)**	5.9%
Positive PRAME (n)	2

**Table 4:** PRAME concordance and molecular concordance for all tested melanocytic lesions.

**Non-challenging melanomas (*n* = 9)**	
Diagnosis: PRAME Concordance (%)	77.8%
Diagnosis: Molecular Concordance (%)	100%
**Diagnostically challenging melanocytic lesions (*n* = 34)**	
Diagnosis: PRAME Concordance (%)	76.5%
Diagnosis: Molecular Concordance (%)	94.1%
**Diagnostically challenging severely atypical nevi (*n* = 26)**	
Diagnosis: PRAME Concordance (%)	96.2%
Diagnosis: Molecular Concordance (%)	100%
**Diagnostically challenging melanomas (*n* = 8)**	
Diagnosis: PRAME Concordance (%)	12.5%
Diagnosis: Molecular concordance (%)	75%

**Table 5:** Diagnostic accuracy measures for PRAME and molecular studies for diagnostically challenging melanocytic lesions.

	**Diagnostically Challenging** **Melanocytic Lesions:** **Prame Staining Results**			**Diagnostically Challenging** **Melanocytic Lesions:** **Copy Number Alteration Results**		
	PRAME +	PRAME −	Total	FISH/SNP +	FISH/SNP −	Total
Treated as Severely Atypical Nevus	1	25	26	0	26	26
Treated as Melanoma	1	7	8	6	2	8
	**PRAME performance** **metrics:** **Diagnostically challenging** **melanocytic lesions**			**Molecular studies performance metrics:** **Diagnostically challenging** **melanocytic lesions**		
Sensitivity	12.50%			75%		
Specificity	96.20%			100%		
PPV	50%			100%		
NPV	78.10%			92.90%		

## Data Availability

The data are not publicly available due to patient privacy concerns. However, upon request and the completion of data transfer agreements, de-identified data are available from the authors. No personal health information will be shared.
